# Right Atrial Thrombus and Submassive Pulmonary Embolism in a COVID-19-Infected Patient: A Case Report

**DOI:** 10.7759/cureus.42221

**Published:** 2023-07-20

**Authors:** Gagandeep Singh Arora, Divya Bhanu Sree Madisetty

**Affiliations:** 1 Internal Medicine, UC (University of California) Riverside, San Bernardino, USA; 2 General Surgery, Government Medical College Patiala, Patiala, IND; 3 Hepatobiliary Pancreatic Surgery and Liver Transplant, BLK-Max Super Speciality Hospital, New Delhi, IND; 4 Internal Medicine, UC (University of California) Riverside School of Medicine, San Bernardino, USA

**Keywords:** cardiac complications, covid-19 complications, thromboembolism, pulmonary embolism, right atrial thrombus, sars-cov-2, covid-19

## Abstract

Coronavirus disease 2019 (COVID-19) is primarily a respiratory infection, but it undoubtedly results in systemic illness that affects multiple systems. The high incidence of thromboembolic events is one distinctive clinical characteristic of COVID-19. This case report is about a unique clinical presentation of a 40-year-old homeless female with polysubstance abuse, who was diagnosed with a right atrial thrombus, sub-massive pulmonary embolism, and COVID-19 infection. The patient presented with shortness of breath, subjective fevers, generalized swelling, and chest and upper abdominal pain. Initially, she was treated with tissue plasminogen activator (TPA) and heparin drip for her thrombi, and she was managed conservatively when hemoptysis ensued post-TPA. She was later sent to a higher level of care for surgical embolectomy. In most cases, severe pulmonary parenchymal disease secondary to COVID-19 correlates with the severity of thromboembolic complications, however, in our case report, there was a right atrial thrombus and pulmonary embolism in the absence of COVID pneumonia. This highlights how notorious COVID-19 infections can be.

## Introduction

The coronavirus disease 2019 (COVID-19) pandemic caused by the severe acute respiratory syndrome coronavirus 2 (SARS-CoV-2) virus presents with a wide range of clinical manifestations but is primarily a respiratory illness [1]. It can affect multiple systems within the body, including the cardiovascular system [2]. Thrombotic complications, such as large vessel strokes, venous thromboembolism, pulmonary embolism, and intracardiac thrombi, have emerged as concerning sequelae in patients with severe COVID-19 [2].

Thrombotic events in COVID-19 are due to a hypercoagulable state induced by the viral infection [2]. Elevated levels of fibrinogen and D-dimer, along with increased inflammatory markers, have been observed in severe cases, indicating an association between the inflammatory response and thrombotic risk [2]. These thrombotic complications can manifest in various clinical presentations, such as pulmonary embolism (PE), which has been reported in COVID-19 patients [2]. In addition to PE, intracardiac thrombi have been reported as well [3-7]. In most cases, the proinflammatory and prothrombotic condition correlates with the severity of the COVID infection and hence with severe COVID pneumonia [8].

This case is of a homeless female patient with a history of polysubstance abuse, who presented with shortness of breath, subjective fevers, and a "puffy" feeling all over her body. Laboratory investigations revealed a positive COVID-19 test result, elevated brain natriuretic peptide (BNP), and a urine drug screen positive for methamphetamines and ecstasy. Imaging studies showed large distal left main pulmonary artery PE extending into the left lower lobe pulmonary artery, bilateral pleural effusions, and a large mobile right atrial thrombus extending from the inferior vena cava into the right atrium. The patient also exhibited systolic heart failure with reduced ejection fraction (HFrEF) on the echocardiogram. Treatment of intracardiac thrombi can be with anticoagulation, fibrinolytic therapy, and/or surgical embolectomy after weighing the risks and benefits of each option [9].

Understanding the interplay between COVID-19, thrombosis, and cardiovascular complications is crucial in effectively managing and improving outcomes. This case report should serve as a reminder of the multifaceted nature of COVID-19 and highlights the importance of a comprehensive approach to managing its various manifestations, including the potentially life-threatening thrombotic complications associated with the disease.

## Case presentation

The patient was a 40-year-old homeless female with a history of polysubstance abuse, including methamphetamines, who presented with symptoms, including shortness of breath, subjective fevers, generalized puffiness, and lower extremity swelling persisting over two weeks. She also complained of a severe, 9/10 sharp and stabbing type of chest pain for the past three days, The pain was sudden in onset, had been progressively worsening, and was exacerbated by deep breathing. She had been enduring the discomfort without any specific measures to alleviate the pain. Ultimately, she decided to seek medical attention.

On physical exam, she was alert and oriented, had rapid shallow breathing, got short of breath while speaking, and had coarse breath sounds bilaterally and bilateral 2+ lower extremity pitting edema. On arrival, she was hypoxic and was started on a non-rebreather mask at 15 liters/minute (l/min), which improved to 5 l/min after initial triage.

A complete blood count showed an elevated white blood cell count of 17,400 cells per microliter suggestive of a possible infection. The hemoglobin and hematocrit were normal at 12.4 grams per deciliter (g/dl) and 40%, respectively. The B-type natriuretic peptide (BNP) levels were significantly elevated at 10,000 picograms per milliliter (pg/ml). Other markers of inflammation and infection were tested, including C-reactive protein (CRP), which was elevated at 18.6 milligrams per deciliter (mg/dl), ferritin at 122.1 nanograms per milliliter (ng/ml), and procalcitonin at 0.20 ng/ml.

An arterial blood gas analysis was performed, revealing a sodium level of 135 millimoles per liter (mmol/L), potassium of 3.9 mmol/L, partial pressure of oxygen (PaO2) of 103 millimeters of mercury (mmHg), partial pressure of carbon dioxide (PaCO_2_) of 24 mmHg, and bicarbonate (HCO_3_) of 14 milliequivalents per liter (mEq/L). This was accompanied by a slightly elevated chloride level at 103 mmol/L and a compensated metabolic acidosis with a pH of 7.4, suggesting possible respiratory alkalosis with an underlying metabolic acidosis.

As part of the infectious workup, cultures were taken from blood, urine, and sputum. Cultures returned negative 48 hours later, indicating that her symptoms were unlikely to be caused by a bacterial infection.

Given her reported substance abuse, a urine drug screen was performed, and came back positive for methamphetamines and ecstasy. Liver function tests were performed due to the patient's reported upper abdominal pain and showed total protein at 6.1 g/dL, albumin at 2.5 g/dL, Aspartate aminotransferase 110 units per liter (U/L), alanine transaminase 174 U/L, alkaline phosphatase 93 U/L, and total bilirubin 1.4 mg/dl, suggesting some form of hepatic dysfunction, possibly related to her substance abuse or congestive hepatopathy. Polymerase chain reaction (PCR) testing revealed a positive COVID-19 test and a urine drug screen indicative of methamphetamine and ecstasy use.

**Figure 1:**
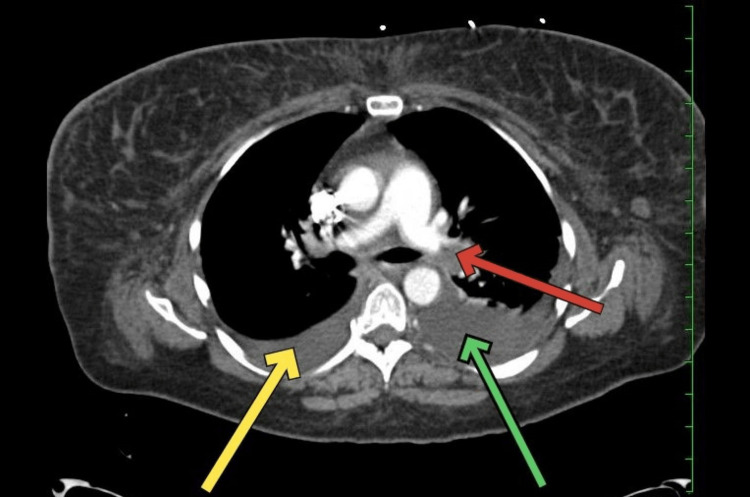
CT pulmonary angiography showing a pulmonary embolus CT pulmonary angiography showing a red arrow pointing at a pulmonary embolus in the left pulmonary artery, a yellow arrow pointing at the right pleural effusion, and a green arrow pointing at the left pleural effusion CT: computed tomography

**Figure 2:**
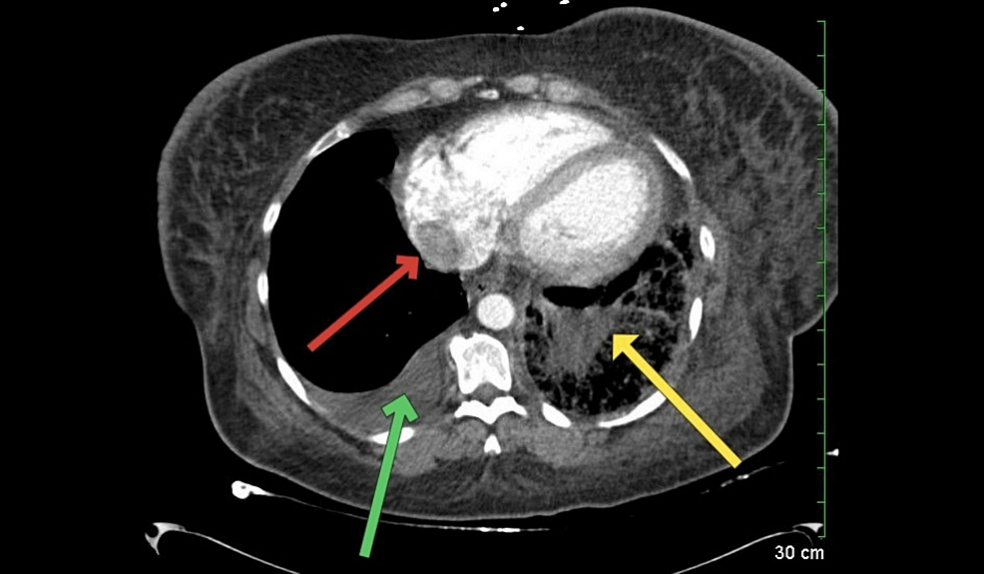
CT pulmonary angiography of the chest CT pulmonary angiography showing a red arrow pointing at the thrombus in the right atrium, a green arrow pointing at the right pleural effusion, and a yellow arrow pointing at a wedge-shaped consolidation in the territory of the pulmonary artery that was blocked by the pulmonary embolus CT: computed tomography

The patient's elevated D-dimer of 35.20 milligrams per liter (mg/L) was concerning for a thrombotic event. A CT chest scan with intravenous contrast was thus performed, which revealed extensive left lower lobe infiltrates, bilateral pleural effusions - free-flowing on the right and loculated on the left, and, most critically, a large distal left main pulmonary artery embolism with no evidence of right heart strain (Figure 1, Figure 2).

**Figure 3:**
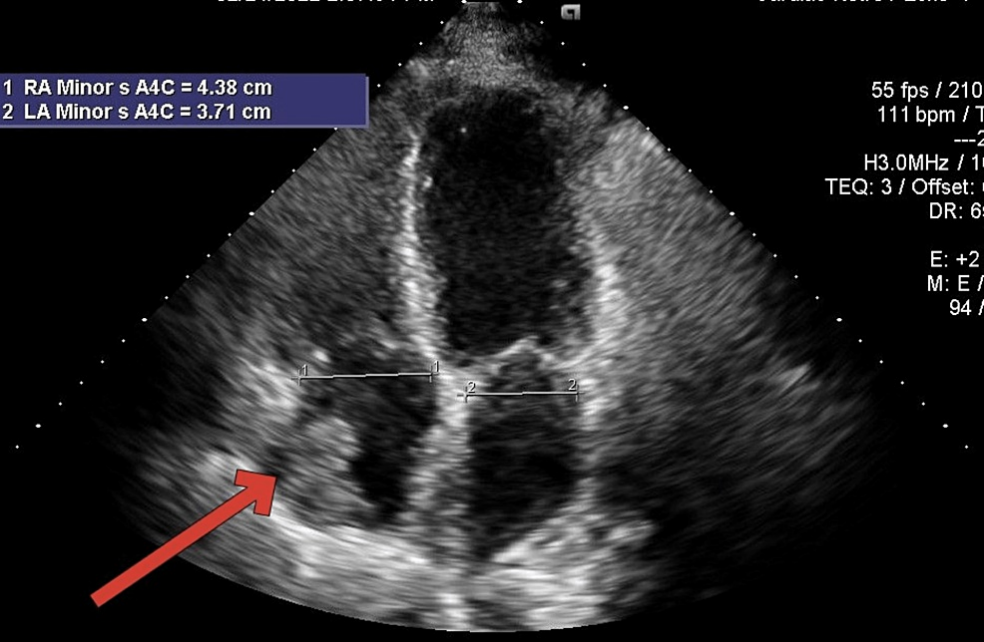
Echocardiogram in the subxiphoid view Echocardiogram in the subxiphoid view showing a red arrow pointing at a thrombus in the right atrium

Duplex ultrasound was also performed on her lower limbs, which ruled out the presence of deep vein thrombosis. Given the discovery of a large pulmonary embolus, a transthoracic echocardiogram was performed to assess the heart's structure and function. This revealed a reduced ejection fraction of 25-30%, a large mobile multilobulated right atrial thrombus measuring 3.6 centimeters (cm) x 2.2 cm extending from the inferior vena cava (IVC), mildly enlarged atria, trace tricuspid regurgitation, and moderately elevated right ventricular systolic pressure of 50 mmHg (Figure 3).

The patient's acute hypoxemia respiratory failure was thought to be due to acute exacerbation of her heart failure and acute pulmonary embolism. Cardiology recommended gentle diuresis with 40 mg IV Lasix once a day. Given the large size of the right atrial thrombus, the patient was considered at high risk for another major thromboembolic event. She was treated with the full dose, i.e. 100 mg intravenous (IV), of tissue plasminogen activator (TPA) given over two hours after undergoing a CT head, which was normal, followed by heparin drip for anticoagulation. Unfortunately, she developed hemoptysis after TPA initiation but was managed conservatively due to stable hemoglobin levels.

Concurrently, she was treated with dexamethasone, remdesivir, and an antioxidant multivitamin, along with IV azithromycin and ceftriaxone for community-acquired pneumonia coverage. On the third day, a repeat echocardiogram revealed no change in the size of the right atrial thrombus although her oxygen requirements had improved and she was at 3 L net negative in fluid balance. Given the risk of an impending major embolism, she was sent to a higher level of care for mechanical thrombectomy and/or surgical embolectomy.

## Discussion

The pathophysiology of COVID-19 and its relation to coagulopathy and thrombosis have been well-discussed in the literature.

COVID-19 is primarily a respiratory illness, which can cause severe pneumonia, acute respiratory distress syndrome (ARDS), and even a cytokine storm in severe cases [1]. The ability of the virus to directly infect endothelial cells leads to endothelial dysfunction and damage to the vascular system [8]. Direct viral infection of endothelial cells and activation of hypoxia-inducible transcription factor-dependent signaling pathway leads to a hypercoagulable state [8].

McFadyen et al. supported this by demonstrating that the thrombotic manifestations of severe COVID-19 are attributed to SARS-CoV-2's ability to invade endothelial cells via ACE-2 receptors expressed on the endothelial cell surface [10]. The hypercoagulable state seen in COVID-19 patients can lead to deep vein thrombosis, stroke, pulmonary embolism, and disseminated intravascular coagulation [2,8,11]. Abnormal coagulation results like elevated D-dimer and fibrin degradation products are linked to a fatal outcome, indicating their potential use in guiding therapy and evaluating prognosis [11]. Wichmann et al. showed that deep venous thrombosis was found in almost half of the autopsies where venous thromboembolism was not suspected before death [12].

There have been conflicting opinions on anticoagulation in COVID patients. Gómez-Mesa et al. showed that low molecular weight heparin as an initial anticoagulant treatment reduced mortality and improved arterial oxygen pressure/inspired fraction of O_2_ (PaO_2_/FiO_2_) [13]. Bikdeli et al. concluded that prophylactic anticoagulation with either low molecular weight heparin (LMWH) or unfractionated heparin (UFH) could prevent venous thromboembolism (VTE) in hospitalized COVID-19 patients with elevated thrombotic risk [14]. They further suggested that anticoagulation should be considered for all hospitalized COVID-19 patients, not just those in the ICU, due to the high risk of thromboembolic events.

Klok et al. underlined the importance of being vigilant for signs of thrombotic complications and strictly applying pharmacological thrombosis prophylaxis in COVID-19 patients admitted to the ICU only [2]. Tang et al. showed that anticoagulant therapy did not significantly reduce mortality in unselected patients with severe COVID-19 [8]. A separate study suggested a trial of anticoagulation in all hospitalized patients with COVID-19 with the use of a higher dose in patients admitted to the ICU [15].

Hanff et al. proposed a more selective strategy that biomarkers of inflammation, coagulopathy, and the renin-angiotensin system pathway should be used to predict the prognosis and severity of COVID-19, to aid clinicians in risk stratification and treatment decision-making [16].

While the most common imaging manifestations of COVID-19 pneumonia are ground-glass opacities and consolidations, the less common but significant findings like the crazy-paving pattern, centrilobular nodular opacities, rounded morphology, cavitation, pleural effusion, and lymphadenopathy, which can sometimes mimic other lung pathologies [17]. 

The most common CT chest findings in acute pulmonary embolism (PE) include emboli presence, pulmonary wedge infarct, pleural effusions, and right heart strain [18]. Thus, differentiating between COVID pneumonia and pulmonary embolism can become tricky at times as COVID-19 by itself increases the chances of thromboembolic phenomena like pulmonary embolism [2], and itself can manifest on the CT scan and interfere with the diagnosis of pulmonary embolism.

Right atrial thrombus with COVID-19 has been reported in isolated case reports [3-7]. Most of these patients had severe COVID pneumonia. Further, with a right atrial thrombus, the overall risk of PE was found to be 100% and in patients with both right and left atrial thrombus, there was an excess PE risk of 250% [19].

Patients with proven or suspected RA thrombus are usually treated with anticoagulants, thrombolytic agents, or surgical thrombectomy, and the choice is determined by weighing the risks and benefits of each [9]. Bigdelian et al. reported an 11-year-old patient with a right atrial thrombus in a COVID- 19 affected patient, where a sternotomy with cardiopulmonary bypass was performed, resulting in the successful removal of the large thrombus while preserving the cardiac valves [3].

In our case, the patient’s respiratory failure was multifactorial due to a submassive pulmonary embolism and suspected reduced ejection fraction heart failure due to cardiomyopathy likely secondary to recreational drug use and was less likely due to COVID-19 pneumonia per se. Moreover, the patient's chest CT scan did not show characteristic diffuse or localized ground-glass opacities and the consolidation seen was in a wedge shape, corresponding to the territory of the left branch of the pulmonary artery, which was blocked by the embolus.

This highlights the issue that COVID-19 can cause a prothrombotic state severe enough to cause a right atrial thrombus or a pulmonary embolus in the absence of severe COVID pneumonia.

## Conclusions

It is crucial to keep in mind that COVID disrupts the equilibrium between pro and anti-thrombotic pathways. For prompt diagnosis and treatment, primary care physicians must maintain a suspicion of thromboembolic events in COVID patients regardless of the severity of the pulmonary parenchymal disease. Echocardiography plays a quick and widely available tool to help in the diagnosis of thromboembolism in COVID patients. Markers of inflammation are associated with a prothrombotic state and the severity of disease, thereby increasing mortality risk in COVID-19. The severity of COVID pneumonia has been linked to a severe prothrombotic state. Our patient is different since although she had a large right atrial thrombus with a submassive pulmonary embolus, she did not have COVID pneumonia features on the CT scan. This highlights the alarming nature of COVID-19, which can manifest in a diverse range of presentations. Anticoagulation treatment must be individualized based on the specific requirements of each patient.
